# Pepsin in Diphtheria

**Published:** 1874-10

**Authors:** 


Use of Pepsin in the Treatment of Diphtheria.—Dr. Doughly advises the following formula for softening and dissolving the false membranes of croup and diphtheria: Pepsin, six grammes; dilute muriatic acid, five drops; distilled water, one hundred grammes. To be used by inhalation, in the same way as lactic acid, as advised by Dr. Weber.—*Revista Clinica di Bologna.*

